# Hydrophobicity and Pore Structure: Unraveling the Critical Factors of Alcohol and Acid Adsorption in Zeolites

**DOI:** 10.3390/molecules29225251

**Published:** 2024-11-06

**Authors:** Yangyang Xie, Honglei Fan, Mingyang Che, Ya Liu, Chunjing Liu, Xin Hu, Botao Teng

**Affiliations:** 1 Tianjin Key Laboratory of Brine Chemical Engineering and Resource Eco-Utilization, College of Chemical Engineering and Materials Science, Tianjin University of Science and Technology, Tianjin 300457, China; a475486151@163.com (Y.X.); fanhonglei@bcig.cn (H.F.); 2 Key Laboratory of the Ministry of Education for Advanced Catalysis Materials, Zhejiang Normal University, Jinhua 321004, China; 13154843717@163.com (M.C.); sky48@zjnu.cn (Y.L.)

**Keywords:** zeolites, hydrophobicity, pore structure, FTS, alcohols and acids

## Abstract

Adsorbing and recycling alcohols and acids from industrial wastewater is of great significance in wastewater treatment; establishing the possible quantitative relationship of alcohol–acid adsorption capacity with the struct0ures of adsorbents and exploring the key factors determining their adsorption performance is very important and challenging in environment science. To solve this difficult problem, the adsorption of C1-5 alcohols, C2-4 acids, and Fischer–Tropsch synthesis (FTS) wastewater on zeolites with similar hydrophobicity and pore structures (β and MFI), similar hydrophilicity but different pore structures (Y and MOR), and similar pore structures but significant differences in hydrophobicity (MOR vs. β and MFI) was systematically investigated. It was found that: (1) For materials with similar pore structures, increased hydrophobicity correlates with enhanced adsorption capacities for alcohols and acids. (2) For materials with similar hydrophobicity, a higher content of ultramicropores leads to increased adsorption of alcohols and acids. (3) Between pore structure and hydrophobicity, it is hydrophobicity that ultimately plays a decisive role in adsorption capacities. The adsorption behavior of zeolites in FTS wastewater exhibits a consistent trend, with β-zeolite demonstrating the highest hydrophobicity (contact angle of 105°) and the greatest adsorption capacity in FTS wastewater, achieving 103 mg/g. Following five adsorption–desorption cycles, the zeolites retained their adsorption capacity without significant degradation, indicating their excellent stability and reusability. The findings identify the critical factors determining adsorption performance and provide a solid foundation for the design and development of high-performance adsorbents for alcohol–acid adsorption.

## 1. Introduction

Low-carbon alcohols and acids are one of the more important and difficult-to-treat pollutants in industrial wastewater. For instance, Fischer–Tropsch synthesis (FTS) [1,2,3] industrial wastewater contains high concentrations of mixed low-carbon alcohols and acids, which not only contribute to environmental pollution but also lead to safety hazards by corroding equipment [4,5]. Therefore, finding efficient and environmentally friendly methods to treat low-carbon alcohol and acid contamination in industrial wastewater is a major challenge in environmental science [6]. Currently, FTS wastewater containing alcohols and acids is typically treated by first neutralizing the low-carbon acids with NaOH [7,8], followed by physical distillation [9,10] to separate low-carbon alcohols from water. The remaining organic pollutants are then treated using biological methods [11,12]. However, this process is lengthy, energy-intensive, and consumes large amounts of NaOH [10,13]. Additionally, the treatment cycle is long, and a large space is required for biological treatment [14,15]. Adsorption methods offer an efficient solution for removing alcohol and acid pollutants from wastewater [16,17,18], reducing contamination of water and soil while allowing for the recovery of valuable low-carbon alcohols and acids [19,20], which has attracted much attention in industrial and academic fields.

The primary factors determining the adsorption capacity of alcohols and acids are the pore structure and surface properties of adsorbents [21,22,23]. Pore structure includes pore size, volume, and distribution [24,25]; surface properties encompass a specific surface area, the type and quantity of surface species, as well as surface hydrophilicity or hydrophobicity, which determine the adsorption performance of adsorbent [26,27]. To elucidate the qualitative and quantitative relationship between these factors and alcohol–acid adsorption capacities, great efforts have been devoted to exploring various adsorbents. Liu et al. [28] systematically studied the relationship between the type and quantity of surface species on graphite, graphene oxide, and reduced graphene with their adsorption alcohol–acid performance. It was found that the presence of COOH, C=O, and C-O groups on graphene surfaces occupied adsorption sites and increased the interaction with water, which was unfavorable for alcohol and acid adsorption; the adsorption capacity was significantly enhanced by reducing the surface oxygen-containing species. However, it is hard to establish a clear qualitative and quantitative relationship between the specific surface functional group and alcohol–acid adsorption capacity due to the complexity and diversity of functional groups on graphene. Zhang et al. [29] systematically studied the adsorption behavior of alcohols and acids on activated carbons with similar hydrophobicity but different pore and surface structures and found that ultramicroporous structures played a decisive role in C1-2 alcohol–acid adsorption; C5 alcohol adsorption was primarily related to specific surface area, while C3-4 alcohol–acid adsorption was determined by ultramicropores and specific surface area. Xie et al. [30] systematically investigated the adsorption of alcohols and acids in carbon nanotubes (CNTs) with different pore distributions and found micropores played a dominant role in the adsorption of alcohols and acids by using chloroform to occupy micropores. Wu et al. [31] found that hydrophobically modified Y/ZSM-5 zeolite composites exhibited excellent adsorption performance for alcohol-containing VOCs and performed well under humid conditions. Edmiston et al. [32] studied hydrophobically modified swellable organically modified silicas (SOMS) and found that the enhanced hydrophobicity of SOMS increased their adsorption capacity for short-chain carboxylic acids (C2-3), but the reduced expandability of modified SOMS led to decreased adsorption capacity for higher carboxylic acids (C4-6). Li et al. [33] provide a molecular perspective on the adsorption behavior of VOC molecules on both neutral and electrically charged CNTs by molecular dynamics simulations, indicating that there is a strong correlation between the adsorption affinity and hydrophobicity of methanol; and VOCs with a higher hydrophobicity demonstrate greater adsorption affinity.

The reported works above have deepened the understanding of alcohol and acid adsorption behavior on adsorbents. However, due to the complex structures of adsorption materials, it is challenging to establish a qualitative and quantitative relationship between pore structure and hydrophilicity/hydrophobicity with alcohol–acid adsorption performance, as modifications of material hydrophilicity/hydrophobicity often alter pore structures simultaneously. To solve this challenge and explore the key factors determining alcohol–acid adsorption capacity, β and MFI zeolites with similar hydrophobicity and pore structures, Y and MOR zeolites with similar hydrophilicity but different pore structures, as well as MOR, β, and MFI zeolites with similar pore structures but significantly different hydrophilicity were selected as model adsorbents. By systematically studying the adsorption behavior of C1-5 alcohols, C2-4 acids, and Fischer–Tropsch wastewater, the qualitative and quantitative relationships of pore structure and hydrophilicity with alcohol–acid adsorption were established. The findings reveal the key factors determining adsorption performance and provide a solid foundation for designing and developing high-performance adsorbents for alcohol–acid adsorption.

## 2. Results and Discussion

### 2.1. Structural Characterization of Zeolites

**Figure 1:**
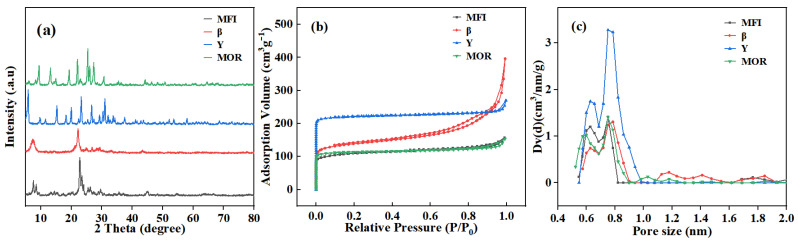
XRD patterns (**a**), N_2_ adsorption and desorption isotherm (**b**), and pore size distribution (**c**) of zeolites.

As shown in Figure 1a, the main diffraction peaks of the four zeolites are observed in the range of 5–35°. The characteristic peaks [34] of MFI appear at 7.86°, 8.76°, 23.24°, and 23.88°, corresponding to (011), (200), (051), and (003) planes, respectively [35]. β zeolite exhibits characteristic peaks [36] at 7.8° and 22.4°, corresponding to (211) and (002) facets, respectively. Y zeolite shows diffraction peaks [37] at 6.24°, 10.16°, 15.70°, 18.73°, 20.41°, and 23.82°, which correspond to (111), (220), (422), (511), (440), and (551) planes of the octahedral Y zeolite, respectively. The diffraction peaks of the MOR zeolite are located [38] at 9.7°, 13.4°, 22.2°, 25.6°, 26.2° and 27.6°, corresponding to (200), (111), (150), (202), (350), and (511) facets, respectively. This is based on the strong diffraction peaks of the four zeolites. The particle sizes calculated by Scherrer’s formula of MFI, β, Y, and MOR zeolites are 15.37, 6.67, 23.59, and 22.02 nm, respectively.

The N_2_ adsorption and desorption isotherms and pore size distribution are shown in Figure 1b,c. To guarantee the reproducibility and accuracy of BET data, the standard sample of MOR zeolite provided by the BET instrument manufacturer was tested. The sample measurements were carried out if the data of the standard sample were consistent with that provided by the instrument productor with an error of 3%. Furthermore, BET data of four zeolites were performed continuously without interruption at the same test conditions. The specific surface area was calculated using the Brunauer–Emmet–Teller (BET) model, and the pore size distribution was determined by the density functional theory (DFT) method. As illustrated in Figure 1b, MFI, Y, and MOR exhibit typical type I adsorption–desorption isotherms with no evident hysteresis loops [39], indicating the characteristic microporous materials. In contrast, β zeolite follows a type I isotherm at low relative pressures but transitions to a type II isotherm with a distinct hysteresis loop when the relative pressure exceeds 0.1, indicating that β is a micro-mesoporous composite material [40]. From Figure 1c and Table 1, the micropore volumes of Y, β, MOR, and MFI zeolites are 0.315, 0.164, 0.154, and 0.133 cm^3^/g, respectively. Y zeolite has the largest ultramicropore (<0.9 nm) volume (0.315 cm^3^/g) and specific surface area (665 m^2^/g), followed by MOR with an ultramicropore volume of 0.141 cm^3^/g and a specific surface area of 430 m^2^/g. Both MFI and β zeolites have an ultramicropore volume of 0.122 cm^3^/g with very similar specific surface areas of 334 m^2^/g and 335 m^2^/g, respectively.

**Figure 2:**
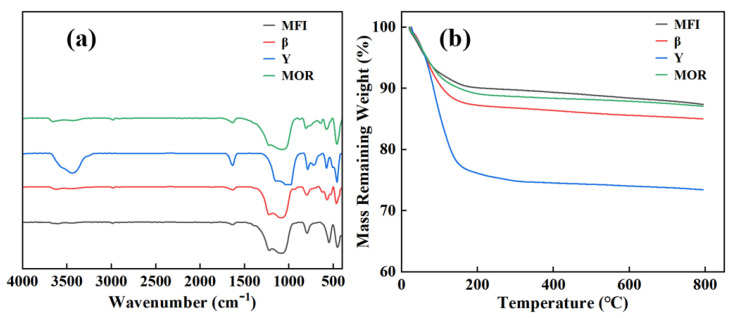
Infrared spectra (**a**) and thermogravimetric analysis of four zeolites under nitrogen purge (**b**).

As shown in the FT-IR spectra of zeolites in Figure 2a, the four types of zeolites exhibit similar IR absorption patterns. The peak at 465 cm⁻^1^ corresponds to the T-O bending vibration of the internal tetrahedral framework, while the peak at 560 cm⁻^1^ is associated with the double-ring vibration. The peaks at 798 and 1230 cm⁻^1^ are attributed to the symmetric and asymmetric stretching vibrations of the external T-O-T linkages, respectively, and the peak at 1085 cm⁻^1^ represents the asymmetric stretching vibration of the internal tetrahedral T-O-T linkages [41]. The peak at 1615 cm⁻^1^ is derived from the bending vibration of lattice water, while the peak at 3475 cm⁻^1^ is assigned to the stretching vibration of adsorbed water. The peak at 3680 cm⁻^1^ indicates the stretching vibration of OH groups [42]. Among these, Y zeolite shows the strongest vibration peaks of water, indicating the highest water content, which will be further corroborated by the thermogravimetric analysis.

As depicted in Figure 2b, all four zeolites exhibit significant weight loss in the range of 50–200 °C, which is attributed to the desorption of physically adsorbed water [43]. Y zeolite shows the highest weight loss, reaching 23%, aligning with its strongest water stretching vibration peaks observed in FT-IR spectra. Between 200 °C and 800 °C, there is almost no further weight loss for zeolites, indicating their excellent thermal stability.

**Figure 3:**
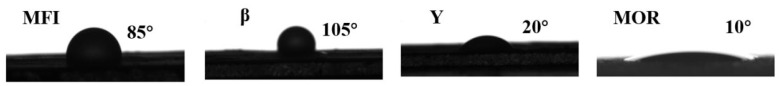
Water contact angle of zeolites.

To investigate the hydrophilic and hydrophobic properties of the four samples, the contact angles were measured and shown in Figure 3. Figure 3 shows that the contact angles of β, MFI, Y, and MOR are 105°, 85°, 20°, and 10°, respectively. This indicates that β is a hydrophobic material; Y and MOR are hydrophilic ones; and MFI is less hydrophilic.

### 2.2. Adsorption Properties of Zeolites

#### 2.2.1. Adsorption of C1-5 Alcohols on Zeolites

**Figure 4:**
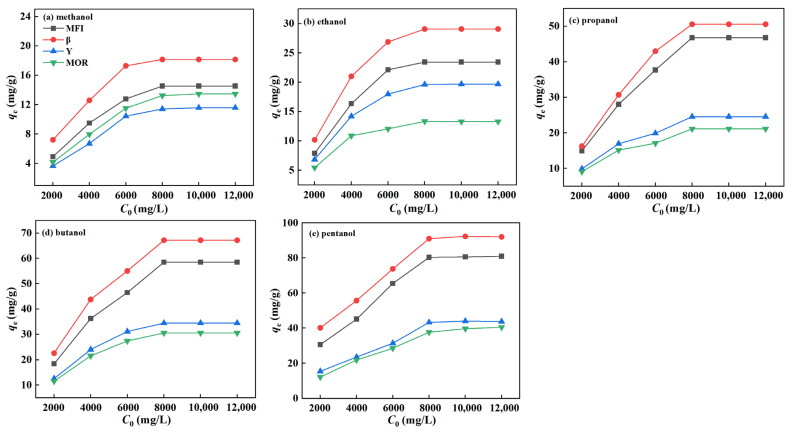
*C*_0_-*q*_e_ curve of zeolites for C1-5 alcohols.

The adsorption isotherms of C1-5 alcohols on zeolites at 25 °C are shown in Figure 4. It was found that the adsorption capacity of zeolites for C1-5 alcohols gradually reaches saturation as the initial concentration increases, with the order of saturation adsorption capacities being β > MFI > MOR ≈ Y. Furthermore, the adsorption capacity increases with the carbon number of alcohols. In Figure 4a,b, the adsorption capacities of β, MFI, MOR, and Y for methanol and ethanol are relatively similar. However, as the carbon chain length increases, in Figure 4c–e, the adsorption capacities of β and MFI for propanol, butanol, and pentanol progressively increase; those of Y and MOR increase at a slower rate, leading to a growing difference in adsorption capacities between the two groups of zeolites.

For β and MFI zeolites, both zeolites share the same ultramicropore volume (0.122 cm^3^/g) despite the significantly larger pore volume of β (0.429 cm^3^/g) compared to MFI (0.197 cm^3^/g). In addition, the contact angle of β is slightly higher than that of MFI zeolite. Therefore, the synergetic effect of hydrophobicity and micropore volume leads to the slightly higher adsorption capacities of alcohols and acids in β zeolite than those in MFI. A similar result was observed in Y and MOR zeolites upon C1-5 alcohol adsorption. Both materials are hydrophilic, with contact angles between 10° and 20°. The larger ultramicropore volume of Y (0.315 cm^3^/g) results in a slightly higher adsorption capacity compared to MOR (0.141 cm^3^/g). This conclusion aligns with the findings by Zhang et al. [29], who reported that C1-5 alcohol adsorption capacities are governed by micropore structure for activated carbon materials with similar hydrophilic/hydrophobic characteristics.

For β and MOR zeolites, the significant difference in contact angles, 105° for β (hydrophobic) and 10° for MOR (hydrophilic), demonstrates the contrasting hydrophilic/hydrophobic properties of these materials. Despite their similar ultramicropore volumes, β exhibits a much higher adsorption capacity for C1-5 alcohols compared to MOR. This suggests that when the ultramicropore structures are comparable, the adsorption capacity of C1-5 alcohols is primarily determined by the material’s hydrophilicity or hydrophobicity. In particular, for the MFI and Y zeolites, although Y has a much larger ultramicropore volume (0.315 cm^3^/g) than MFI (0.122 cm^3^/g), MFI’s higher contact angle (85° vs. 20° for Y) results in MFI having a greater adsorption capacity for C1-5 alcohols, especially for C3-5 alcohols. Therefore, the hydrophilicity/hydrophobicity plays a more critical role in determining the adsorption of C1-5 alcohols than the micropore volume. This is due to the competitive adsorption of water and alcohols, leading to the low adsorption capacities of hydrophilic zeolites (MOR and Y) and the high performance of hydrophobic zeolites (β and MFI).

#### 2.2.2. Adsorption of C2-4 Acids on Zeolites

**Figure 5:**
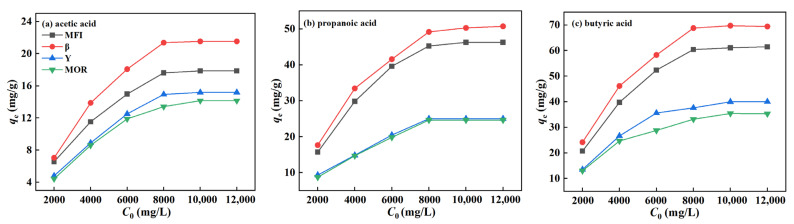
*C*_0_-*q*_e_ curve of zeolites for C2-4 acids.

From the adsorption isotherms of C2-4 acids on zeolites in Figure 5, it is found that the adsorption behavior of zeolites for low-carbon acids follows a pattern similar to that for alcohols. As the initial concentration increases, the adsorption of low-carbon acids gradually reaches saturation, with the order of adsorption capacity being β > MFI > MOR ≈ Y. Additionally, as the carbon chain length increases, the adsorption capacity for C2–C4 acids also increases. For propionic acid and butyric acid, the adsorption capacities of β and MFI are quite similar, while Y and MOR zeolites exhibit significantly lower adsorption capacities for low-carbon acids compared to β and MFI due to the competitive adsorption between water and acids. Moreover, the difference in adsorption capacities between these groups becomes more pronounced as the carbon number increases.

This result can be attributed to factors of the hydrophilic/hydrophobic properties and pore structure of zeolites. The higher contact angles of β and MFI zeolites, which are significantly greater than those of Y and MOR zeolites, are the primary reason for their much higher adsorption capacities. Similarly, despite Y having a much larger ultramicropore volume (0.315 cm^3^/g) than MOR (0.141 cm^3^/g), their adsorption capacities for C2-4 acids are very similar due to their comparable and relatively small contact angles. Thus, for low-carbon acid adsorption, the hydrophilicity/hydrophobicity of zeolites remains the key factor determining their performance.

#### 2.2.3. Adsorption Isotherm Data Fitting of Single Alcohol and Acid

**Figure 6:**
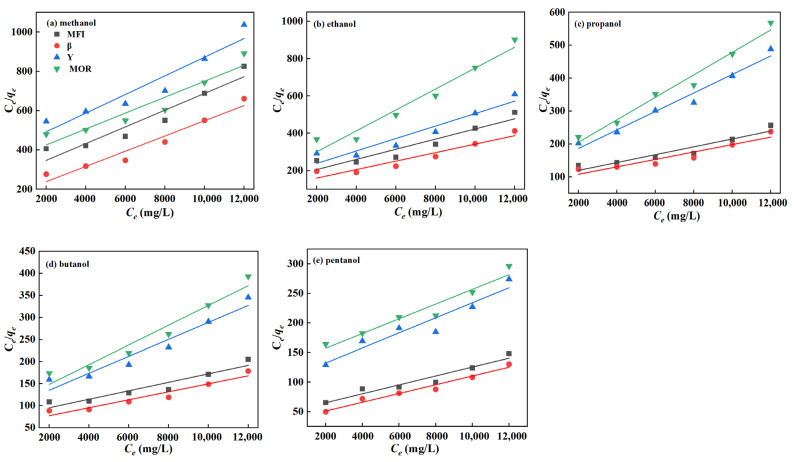
Langmuir fitting curve of C1-5 alcohols on zeolites.

**Figure 7:**
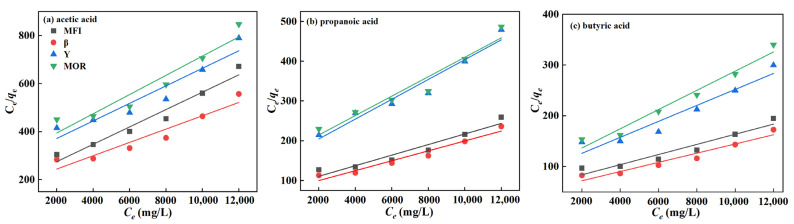
Langmuir fitting curve of C2-4 acids on zeolites.

To gain a deeper understanding of the adsorption behavior of C1-5 alcohols and C2–C4 acids on zeolites, Langmuir and Freundlich adsorption isotherm models were employed for fitting the data, as shown in Figure 6 and Figure 7. The corresponding parameters are listed in Table 2 and Table 3.

From Figure 6 and Figure 7 and Table 2 and Table 3, it is found that the linear correlation coefficients (R^2^) of the Langmuir isotherm model are all greater than 0.9, while those of the Freundlich isotherm model are all less than those of the Langmuir model by 0.03–0.17. In addition, the pores of four zeolites are too small to accumulate multiple molecules side by side for adsorption at the same time. Hence, alcohols and acids only adsorb one by one in the zeolite pore, which is similar to the monolayer adsorption on the uniform surface of the Langmuir model. However, the Freundlich model is suitable for multilayer adsorption for a non-uniform surface. Moreover, the maximum adsorption capacities of β, MFI, Y, and MOR zeolites for alcohols and acids, as predicted by the Langmuir model, are consistent with the experimental values. For instance, the maximum adsorption capacities for pentanol are 93, 82, 44, and 41 mg/g, respectively, which closely match the experimental values of 91, 80, 40, and 38 mg/g. Therefore, the Langmuir adsorption model is preferred in this work for the adsorption of alcohol and acid molecules on zeolites. Given the small external specific surface area of the zeolites, it is inferred that the alcohol and acid molecules are primarily adsorbed within the micropores of zeolites.

#### 2.2.4. Adsorption of Fischer–Tropsch Synthetic Wastewater

**Figure 8:**
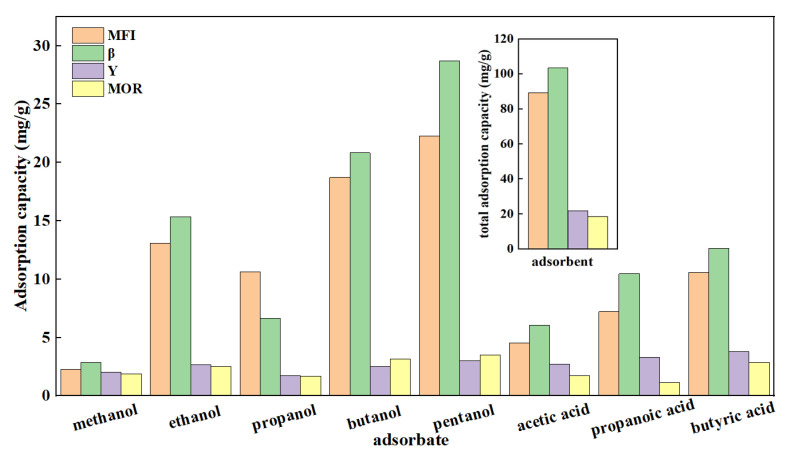
Adsorption effect of molecular sieves on FTS zeolites.

To investigate the effects of different pore structures and hydrophilicity of zeolites on the adsorption of alcohols and acids in Fischer–Tropsch synthesis wastewater, the adsorption performance of zeolites for FTS modeling wastewater was systematically measured, as shown in Figure 8. Due to the strong hydrophilicity of Y and MOR zeolites, their adsorption capacities for C1-5 alcohols and C2-4 acids in FTS wastewater were relatively low, leading to the low alcohol–acid adsorption capacities of 21.82 mg/g and 18.53 mg/g, respectively. In contrast, β and MFI zeolites exhibit weaker hydrophilicity, resulting in significantly higher adsorption capacities for C1-5 alcohols, C2-4 acids, and total alcohols and acids in FTS wastewater at 103.52 mg/g and 89.27 mg/g, respectively. This result aligns well with their performances in pure alcohol and acid adsorption. That is, for zeolites with similar ultramicropore structures, higher hydrophobicity leads to higher alcohol and acid adsorption; for zeolites with similar hydrophilicity, a higher ultramicropore content results in greater alcohol and acid adsorption. Between ultramicropore content and hydrophilicity, the latter plays a decisive role in alcohol and acid adsorption.

#### 2.2.5. Zeolite Regeneration Experiment

**Figure 9:**
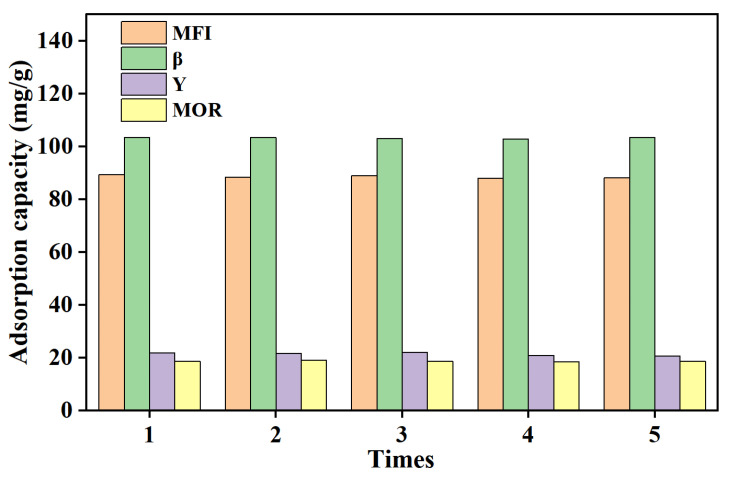
Regeneration experiment of zeolites.

After the adsorption of alcohols and acids from FTS wastewater, the zeolites were separated by centrifugation and dried overnight at 120 °C. The regenerated zeolites were then used in repeatability experiments at 25 °C. As shown in Figure 9, the adsorption performance of zeolites remained largely unchanged after five adsorption–desorption cycles, indicating their good stability and renewability.

## 3. Experiment

### 3.1. Reagents

MFI, β, Y, and MOR zeolites were purchased from Kaimat (Tianjin) Chemical Technology Co., Ltd. (Tianjin, China). Methanol, ethanol, propanol, butanol, pentanol, acetic acid, propionic acid, and butyric acid used for preparing FTS wastewater were all analytical agents and obtained from Aladdin Reagent Co., Ltd. (Shanghai, China).

### 3.2. Characterization

The crystal structure of zeolites was analyzed using a PHILIPS PW3040/60 X-ray powder diffractometer (XRD) with Cu Kα radiation (Philips, Amsterdam, The Netherlands), scanning from 5° to 80° at 5°/min. The hydrophilicity and hydrophobicity of samples were measured using a KRUSS DSA30 contact angle meter (KRÜSS Scientific, Hamburg, Germany). The samples were pretreated under vacuum at 150 °C for 6 h to remove impurities, then pore structure and specific surface area were determined by N_2_ adsorption–desorption measurements at −196 °C using a Bayside physical adsorption instrument (BSD-PM, Beijing, China). Thermogravimetric (TG) and differential thermal analysis (DTA) curves were obtained with a TA Instruments thermogravimetric analyzer (SDT-Q600) by heating the samples from room temperature to 800 °C under a nitrogen atmosphere at a heating rate of 10 °C/min (TA Instruments, New Castle, DE, USA). FT-IR spectra of zeolites were performed by infrared spectroscopy (NICOLET 6700, Thermo Fisher Scientific, Waltham, MA, USA).

### 3.3. Adsorption Measurement

The adsorption capacities of different zeolites for C1-5 alcohols, C2-4 acids, and Fischer–Tropsch synthesis (FTS) wastewater were measured at 25 °C. Accurately measure the required amount of alcohol or acid using a pipette and transfer it into a 250 mL volumetric flask. Then, dilute to volume to obtain the required concentration ranging from 2000 to 12,000 mg/L and shake well for later use. The FTS modeling wastewater concentrations of C1–C5 alcohols and C2–C4 acids are listed in Table 4. For each experiment, 1 g of zeolite was placed into a 250 mL conical flask, followed by the addition of 100 mL of alcohol, acid solution, or FTS wastewater. After sealing and thoroughly mixing, the flask was placed in a thermostatic air bath shaker at 25 °C and shaken at 120 rpm for 2 h. The solution was then filtered through a 0.45 μm organic membrane filter, and the filtrate was analyzed using a gas chromatograph (Agilent 8890, Agilent Technologies, Santa Clara, CA, USA) to determine the concentrations of the adsorbate components.

## 4. Conclusions

Based on the adsorption studies of C1-5 alcohols, C2-4 acids, and Fischer–Tropsch synthesis wastewater on zeolites with similar hydrophobicity and pore structures (β and MFI), similar hydrophilicity but different pore structures (Y and MOR), and similar pore structures but significant differences in hydrophobicity (MOR vs. β and MFI), the results indicate that: (1) with the similar pore structure, higher hydrophobicity leads to greater alcohol and acid adsorption capacity; (2) with the similar hydrophobicity, higher ultramicropore content results in greater alcohol and acid adsorption; (3) between the two factors of pore structure and hydrophobicity, it is hydrophobicity that ultimately plays the determining role. The adsorption behavior of zeolites in FTS wastewater follows the same pattern, with β-zeolite showing the highest hydrophobicity (contact angle of 105°) and the highest adsorption capacity for alcohols and acids in FTS wastewater, reaching 103 mg/g. After five adsorption–desorption cycles, the zeolites maintained their adsorption capacity without significant degradation, demonstrating their high stability and reusability.

## Figures and Tables

**Table 1:** BET areas and pore structures.

Sample	*S*_BET_ (m^2^/g)	*V*_0.9_ (cm^3^/g)	*V*_0.9–2_ (cm^3^/g)	*V*_t_ (cm^3^/g)
MFI	334	0.122	0.011	0.197
β	430	0.122	0.042	0.429
Y	665	0.315	0	0.335
MOR	335	0.141	0.013	0.195

*S*_BET_: specific surface of activated zeolites when P/P_0_ is in the range of 0.01–0.1 (Brunauer–Emmet–Teller); *V* < 2 nm: pore volume with a pore size less than 2 nm (DFT method); *V*_t_: total pore volume calculated when P/P_0_ = 0.99 (DFT method).

**Table 2:** Langmuir and Freundlich fitting parameters of C1-5 alcohols on zeolites.

Adsorbate	Sample	Langmuir			Freundlich		
		*R* ^2^	*q*_m_	*K*_1_	*R* ^2^	*n*	*K*_2_
methanol	MFI	0.92	14.57	0.0426	0.88	2.03	0.1573
	β	0.95	18.20	0.0387	0.85	2.35	0.3661
	Y	0.91	11.60	0.0478	0.87	1.87	0.0847
	MOR	0.91	13.50	0.0405	0.89	1.84	0.0891
ethanol	MFI	0.90	23.51	0.0273	0.83	2.16	0.3377
	β	0.91	29.20	0.0228	0.84	2.23	0.4778
	Y	0.97	19.74	0.0334	0.85	2.21	0.3120
	MOR	0.98	13.32	0.0561	0.82	2.64	0.4106
propanol	MFI	0.91	47.13	0.0120	0.89	1.83	0.3005
	β	0.90	50.95	0.0113	0.89	1.88	0.3794
	Y	0.97	24.64	0.0281	0.88	2.22	0.3832
	MOR	0.97	21.19	0.0341	0.89	2.36	0.4274
butanol	MFI	0.91	59.01	0.0096	0.88	1.84	0.3908
	β	0.93	67.80	0.0091	0.89	1.97	0.6232
	NaY	0.94	34.88	0.0192	0.87	2.21	0.5387
	MOR	0.95	30.33	0.0224	0.87	2.26	0.5206
pentanol	MFI	0.94	81.91	0.0075	0.89	1.89	0.6231
	β	0.97	93.10	0.0074	0.89	2.13	1.2130
	Y	0.92	44.07	0.0127	0.88	1.72	0.2039
	MOR	0.94	40.79	0.0125	0.89	1.63	0.1399

**Table 3:** Langmuir and Freundlich fitting parameters of C2-4 acids on zeolites.

Adsorbate	Sample	Langmuir			Freundlich		
		*R* ^2^	*q*_m_	*K*_1_	*R* ^2^	*n*	*K*_2_
acetic acid	MFI	0.96	17.86	0.0361	0.89	2.06	0.2018
	β	0.92	21.53	0.0277	0.89	1.95	0.1920
	Y	0.95	15.19	0.0365	0.88	1.75	0.0787
	MOR	0.92	14.16	0.0400	0.89	1.81	0.0871
propanoic acid	MFI	0.93	46.28	0.0133	0.87	1.99	0.4576
	β	0.95	50.74	0.0125	0.89	2.02	0.5255
	Y	0.95	25.01	0.0249	0.89	1.95	0.2232
	MOR	0.94	24.64	0.0244	0.89	1.90	0.1917
butyric acid	MFI	0.93	61.53	0.0100	0.89	1.99	0.6041
	β	0.94	69.43	0.0091	0.87	2.04	0.7529
	Y	0.93	40.02	0.0157	0.88	2.06	0.4561
	MOR	0.97	35.28	0.0189	0.89	2.14	0.4706

**Table 4:** The concentrations of FTS modeling wastewater.

Adsorbate	Concentration (mg/L)	Adsorbate	Concentration (mg/L)
methanol	5110	pentanol	617
ethanol	7907	acetic acid	4225
propanol	1942	propanoic acid	842
butanol	1125	butyric acid	450

## Data Availability

The data presented in this study are available on request from the corresponding author.
